# Clinical effect of channel assisted cervical key hole technology combined with ultrasonic bone osteotome in the treatment of single segment cervical spondylotic radiculopathy

**DOI:** 10.3389/fsurg.2022.1029028

**Published:** 2022-10-17

**Authors:** Junlin Liu, Qingquan Kong, Pin Feng, Bin Zhang, Yuan Hu, Junsong Ma

**Affiliations:** ^1^ Department of Orthopedics Surgery, Hospital of Chengdu Office of People's Government of Tibetan Autonomous Reigion, Chengu Sichuan; ^2^ West China Hospital, Sichuan University, Chengdu, China

**Keywords:** key hole, ultrasonic bone osteotome, cervical spondylotic radiculopathy, clinical effect, operating skills

## Abstract

**Objective:**

To explore the clinical effect and operating skills of channel assisted Cervical Key Hole technology combined with Ultrasonic Bone Osteotome (CKH-UBO) in the treatment of single segment cervical spondylotic radiculopathy (CSR).

**Methods:**

From June 2018 to June 2020, 14 patients diagnosed with CSR and treated with channel assisted CKH-UBO were collected. The duration of the disease, the length of the incision, the operation time, the amount of bleeding during the operation, the length of hospitalization and the complications were recorded. The Range Of Motion (ROM) and the stability of the surgical segment were recorded before and after the operation. Visual analog scale (VAS), neck disability index (NDI) and modified macnab efficacy evaluation criteria were used to evaluate the surgical efficacy.

**Results:**

The operative segments of the enrolled patients were all lower cervical vertebrae. The average incision length was 2.0 ± 0.1 cm, the operation time was 42.2 ± 5.7 min, the intraoperative bleeding volume was 32.7 ± 4.1 ml, and the hospital stay was 5.6 ± 1.2 days. There was no difference in ROM between preoperative and 3 months and 1 year after operation (*P* > 0.05), and all patients did not have segmental instability. The VAS scores of neck pain before surgery, 3 days after surgery, 3 months after surgery, and 1 year after surgery were 5.6 ± 1.2, 1.6 ± 0.6, 1.1 ± 0.7, 0.6 ± 0.5, and the VAS scores of upper limb pain were 6.2 ± 1.2, 1.7 ± 0.7, 1.1 ± 0.6, 0.6 ± 0.5. The NDI scores of upper limb pain before surgery, 3 days after surgery, 3 months after surgery, and 1 year after surgery were 36.7 ± 3.5, 9.8 ± 2.4, and 3.9 ± 1.5, 1.8 ± 1.0, The VAS and NDI scores at all follow-up time points after operation were significantly lower than those before operation (*P* < 0.001). One year after operation, the curative effect was evaluated according to the modified macnab evaluation standard, and the excellent and good rate was 100%. The complication rate was 6.25%.

**Conclusion:**

Channel assisted CKH-UBO for single segment CSR has the advantages of short operation time, reliable clinical effect, high safety and low complication rate, which is worthy of clinical promotion.

## Introduction

CSR needs surgical treatment after conservative treatment is ineffective. Anterior cervical fixation and fusion is a classic surgical method for CSR treatment. However, after fixation and fusion, it will affect the cervical mobility unit of the responsible segment and accelerate the degeneration of adjacent segments (1, 2). In recent years, with the rapid development of minimally invasive spine, minimally invasive key hole surgery has been gradually applied to cervical spine surgery. A large number of literatures have reported that key hole endoscopic surgery can obtain good clinical efficacy (3, 4). The full resection of bone structures and the exposure of nerves are the key steps of this operation. However, the interference of soft tissue on the visual field under the microscope and the poor hemostatic technology under the microscope will reduce the operation efficiency. Meanwhile, when the grinding drill is used for bone cutting under the microscope, the scraping effect of the grinding drill is often prone to complications such as dural tear and nerve root injury, therefore, it has high technical requirements for the operator and is not suitable for beginners, which also reduces the practicability of the operation (5). With the advent of ultrasonic osteotome and its gradual application in orthopedics since 1998 (6), many scholars found that ultrasonic osteotome has more advantages in orthopedic surgery. Compared with grinding drill, ultrasonic osteotome is more efficient, stable and safe (7–9). Early literatures reported the use of ultrasonic osteotome under endoscope, but due to the difficulty of operation under endoscope and the choice of knife head, limited choice does not provide obvious convenience for surgery (10). The channel assisted cervical key hole surgery allows the use of conventional open surgical instruments, which provides sufficient space and conditions for the use of ultrasonic osteotomes. Therefore, our team tried to apply the ultrasonic osteotome to the channel assisted key hole operation of cervical spine 4 years ago and accumulated some application experience. Therefore, we conducted a retrospective analysis on this group of patients to explore the clinical efficacy and operation skills of CKH-UBO in the treatment of CSR.

## Methods and materials

### Patients selection

This study is a retrospective study. The study protocol was approved by the hospital ethics committee and carried out according to the declaration of Helsinki. A total of 14 patients diagnosed with CSR and treated with channel assisted CKH-UBO between June 2018 and June 2020 were included. Inclusion criteria: (1) Definite diagnosis of single segment cervical spondylotic radiculopathy; (2) All patients had unilateral symptoms and underwent unilateral decompression; (3) The lesion site was lower cervical vertebra (C3-C7); (4) Intraoperatively, UBO was used for fenestration and decompression; (5) After standard conservative treatment, the effect is poor or the symptoms are progressive. Exclusion criteria: (1) Revision surgery; (2) Imaging showed that the cervical spine was unstable; (3) The number of decompression sections exceeds 1; (4) There were ossification and osteophyte on the ventral side of the nerve; (5) Failure to follow up as planned; (6) Persons with mental illness or mental disorder; (7) Presence of intracranial or peripheral neuropathy; (8) There are contraindications or no surgical treatment.

### Surgical technique

The patients lie prone on the spinal operation mattress, fix the head in the flexion position with Macintosh's head rest, fluoroscopically locate the surgical segment and mark it. All patients use neuroelectrophysiological monitoring. After routine disinfection and towel laying, the intraoperative operation was started. The skin, subcutaneous tissue and deep fascia layer were cut by 10 mm beside the midline, and the soft tissue was separated and expanded by using a step-by-step expansion tube. Finally, a non expanded fixed channel with an internal opening of 20 mm and an external opening of 24 mm was placed. The fixed channel of the free arm was connected to the appropriate position. After the fluoroscopy of the C arm was clear and the position was satisfactory, the decompression operation was performed. Use an electric knife to strip the residual soft tissue on the bone surface, identify the vertebral lamina and lateral mass, and expose the V-point. The lower edge of the upper lamina and the upper edge of the lower lamina were gradually scraped with a spoon shaped UBO. Carefully separate and bite the ligamentum flavum with a lamina osteotome. Fully expose the outer edge of the spinal cord and the suprashoulder and axillary areas of the nerve root, and expand the decompression range according to the decompression plan prepared before the operation. Separate the nerve root, expose the prominent nucleus pulposus tissue, move gently and pay attention to fully protect the nerve. Use nucleus pulposus forceps to remove the protruding nucleus pulposus tissue, and use bipolar electrocoagulation, gelatin sponge and other hemostatic materials to fully stop bleeding after the nerve decompression is fully explored. The channels were removed and hemostatic sutures were performed layer by layer. All the patients in the group did not place a drainage tube after the operation. On the second day after the operation, they were re examined by imaging and moved with the assistance of a neck brace. On the 3rd–5th day after the operation, the patient can be discharged from the hospital if the incision is normal, and continue to standardize functional exercise. One month after the operation, the neck brace can be removed and the normal neck movement can be restored.

### Outcome measures

General data such as gender, age, length of surgical incision, operation time, intraoperative blood loss and hospitalization time of the enrolled patients were recorded. The influence of channel assisted CKH-UBO on the local stability of the cervical spine was evaluated by recording the ROM according to penning method (11) before and after operation and the stability of the operative segments with dynamic radiographs of cervical flexion and extension. Criteria for cervical instability: the x-ray film of flexion and extension dynamic position shows that the sagittal displacement is >3.5 mm and the angular displacement is >11° (12). VAS, NDI and modified macnab evaluation criteria were used to evaluate the surgical effect. The incidence and types of complications were recorded to evaluate the safety of the operation.

### Statistical analysis

SPSS 20.0 statistical software was used for data analysis. The measurement data were expressed as mean ± standard deviation. If the data conformed to normal distribution, paired t test was used for preoperative and postoperative continuity data comparison. If the data did not conform to normal distribution, paired Wilcoxon rank sum test was used for analysis. The inspection level is taken from both sides *α* = 0.05.

## Results

### Demographics characteristics

A total of 14 patients were included in this study, including 11 males and 3 females, with an age of 51.1 ± 8.4 years and a course of 16.9 ± 7.5 months. The operation sites were all lower cervical vertebrae, 3 cases were C4/5, 7 cases were C5/6 and 4 cases were C6/7. All patients underwent single segment channel assisted CKH-UBO surgery, and the decompression mode was unilateral approach and unilateral decompression. The average incision length was 2.0 ± 0.1 cm, the operation time was 42.2 ± 5.7 min, the intraoperative bleeding volume was 32.7 ± 4.1 ml, and the hospital stay was 5.6 ± 1.2 days (Table 1).

**Table 1:** Summary of the baseline data.

Characteristics	CKH-UBO (*n* = 14)
Age (years)	51.1 ± 8.4
Sex M/F	11/3
Duration of symptoms (months)	16.9 ± 7.5
Surgical location	
C4/5	3
C5/6	7
C6/7	4
Operating time (min)	42.2 ± 5.7
Blood loss (ml)	32.7 ± 4.1
Incision length (cm)	2.0 ± 0.1
Hospital stay (d)	5.6 ± 1.2

CKH-UBO indicates Cervical Key Hole-Ultrasonic Bone Osteotome; *n* indicates the total number of patients.

### Radiological results

During the follow-up of 3 months and 1 year after operation, none of the enrolled patients showed segmental instability. The ROM of cervical spine measured by preoperative dynamic position x-ray film was 51.3 ± 3.1°, and the ROM of cervical spine was 50.1 ± 2.6°, 51.2 ± 3.5° at 3 months and 1 year after operation. There was no statistical difference between postoperative and preoperative (Table 2).

**Table 2:** Comparation of the ROM between pre and postoperative.

Characteristics	Pre-op	Post 3m-op	Post 1y-op
ROM	51.3 ± 3.1*	50.1 ± 2.6^/^	51.2 ± 3.5^#^

ROM indicates Range of motion.

The scoring system is used to assess lumbar spine stability. *P* = 0.387 if ^/^ is compared with *, *P* = 0.833 if ^#^ is compared with *.

### Functional results

The VAS score of neck pain before surgery, 3 days after surgery, 3 months after surgery, and 1 year after surgery were 5.6 ± 1.2, 1.6 ± 0.6, 1.1 ± 0.7 and 0.6 ± 0.5. The VAS score of upper limb pain before surgery, 3 days after surgery, 3 months after surgery, and 1 year after surgery were 6.2 ± 1.2, 1.7 ± 0.7, 1.1 ± 0.6 and 0.6 ± 0.5. The NDI score before surgery, 3 days after surgery, 3 months after surgery, and 1 year after surgery were 36.7 ± 3.5, and 9.8 ± 2.4, 3.9 ± 1.5 and 1.8 ± 1.0. The VAS score and NDI score at each follow-up time point after operation were significantly improved compared with those before operation, and the difference was statistically significant. After 1 year follow-up, according to the modified macnab evaluation standard, 9 cases were excellent, 5 cases were good, 0 case was fair, 0 case was poor, and the excellent and good rate was 100% (Table 3).

**Table 3:** Comparation of the functional score between pre and postoperative.

Characteristics	Pre-op	Post 3d-op	Post 3m-op	Post 1y-op
VAS neck	5.6 ± 1.2*	1.6 ± 0.6^/^	1.1 ± 0.7^/^	0.6 ± 0.5^/^
VAS upper limb	6.2 ± 1.2^#^	1.7 ± 0.7^%^	1.1 ± 0.6^%^	0.6 ± 0.5^%^
NDI	36.7 ± 3.5^$^	9.8 ± 2.4^^^	3.9 ± 1.5^^^	1.8 ± 1.0^^^
Macnab				
Excellent				9
Good				5
Fair				0
Poor				0
E and G Rate				100%

VAS indicates Visual Analogue Scale; NDI indicates Neck Disabilitv Index; E indicates Excellent; G indicates Good; pre-op indicates preoperative; post-op indicates postoperative.

*P* < 0.001 if ^/^ is compared with *, *P* < 0.001 if % is compared with ^#^, *P* < 0.001 if ^^^ is compared with ^$^.

### Complications

**Figure 1:**
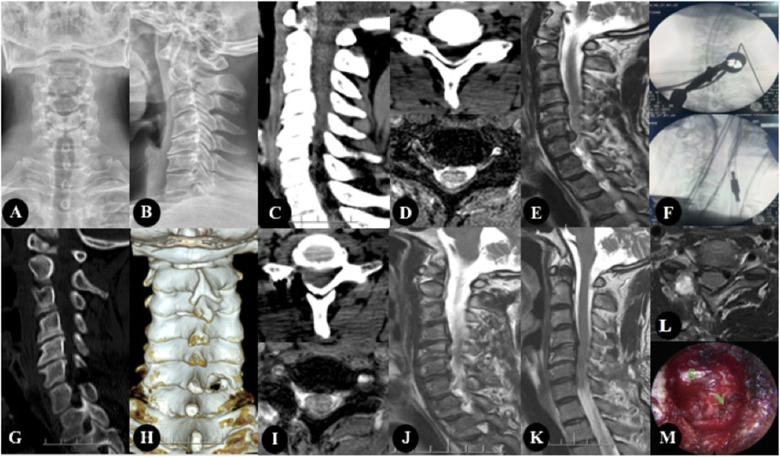
The patient, Male, 47 years old, was diagnosed with CSR(C6/7). (**A–E**) Preoperative imaging studies. (**A,B**) showed cervical kyphosis, without rotation, slippage and scoliosis. (**C,D**) showed there are osteophytes at the posterior edge of the vertebral body, and the intervertebral foramen area is not narrow. (**D,E**) showed cervical intervertebral disc protrusion, located in the right foramen region. (**F**) showed Intraoperative fluoroscopy. (**G–L**) were the postoperative imaging examination, in which (**G–J**) were the re-examination data 3 days after the operation, and (**K,L**) were the re-examination data 1 year after the operation. (**G,I**) were CT images 3 days after operation, and The facet joints were well preserved and the range of fenestration was satisfactory. (**I,J**) showed that the nucleus pulposus in the right foramen area was completely removed. (**K,L**) showed there was no recurrence 1 year after operation. (**M**) showed intraoperative image.

One patient developed numbness of the affected side of the forearm after the operation, and completely disappeared after symptomatic treatment such as nutritional nerve. All patients had no nerve injury, dural sac tear, cerebrospinal fluid leakage, infection, intraspinal hematoma, etc. none of the patients had relapse after 1-year follow-up (Figure 1).

## Discussion

### Effectiveness of channel assisted CKH-UBO

In terms of osteotomy efficiency. The key hole operation under endoscope uses grinding drill to remove the bone structure, and the operation mode is shallow to deep layer by layer grinding, which is inefficient (13). In the process of grinding and removing the bone structure, it is necessary to constantly identify the anatomical marks to ensure the scope of the bone structure removal. These operations may increase the operation time. In addition, when the bone structure is removed under endoscope, the surrounding soft tissue often blocks the visual field, and the position of the endoscope needs to be adjusted frequently to expose the bone structure, which also leads to the reduction of the operation efficiency. The key hole operation under the channel significantly expanded the operation field and operation space. Under the channel, electric scalpels and surgical tools of the same size as open surgery can be used to quickly remove the soft tissues around the facet joints and the vertebral lamina margin, and it is clear, which creates favorable conditions for the resection of bony structures. In addition, the use of UBO under the channel can improve the efficiency of bone structure resection, which is also the key to shorten the operation time. We believe that the high efficiency of UBO is mainly reflected in the following points. First of all, the blade head we use is spoon-shaped. Its function is similar to that of an electric scraper, which is different from that of a grinding drill, the UBO can directly scrape off the vertebral lamina without layered grinding. Secondly, the UBO is mainly used to cut the bone through its own micro vibration (14). The vibration range is only at the tip of the knife head, and the controllability is strong. Therefore, the operator can operate with one hand, and the other hand can use the attractor to keep the visual field of the operation area clear, reducing part of the time spent on replacing the surgical instruments. In this group of experiments, the average operation time was 42.2 ± 5.7 min, which was not increased compared with the operation time of endoscopic key hole technology reported in other literatures (15–17). This also shows that the channel assisted CKH-UBO operation is time-consuming and efficient.

In terms of clinical efficacy. Due to the limited field of vision of the key hole under endoscope, it may lead to misjudgment of the decompression range, which may further affect the clinical efficacy. Although domestic and foreign literatures have reported many methods for defining the decompression range, they can not accurately judge the decompression boundary under direct vision (18–20). However, the channel assisted method was adopted in this group of experiments, which can display the anatomical structure more widely and clearly than endoscopy, providing a prerequisite for accurate decompression. All the enrolled patients fully exposed the supra shoulder and axillary regions of the nerve roots, which ensured the adequacy of decompression during the operation. In addition, the UBO can accurately reach the decompression target. Because its force direction is dorsal to the nerve, this operation characteristic reduces the interference of the surgical operation on the nerve, thus avoiding the influence of the clinical effect due to the nerve stimulation symptoms after the operation. In addition, for some patients with intervertebral foramen stenosis, UBO can also safely and efficiently decompress the region. The postoperative NDI score and VAS score of the patients in this group were significantly lower than those before the operation. The excellent and good rate of modified macnab was 100%. The above statistical results indicate that the clinical efficacy of channel assisted CKH-UBO is reliable.

### Safety of channel assisted CKH-UBO

In terms of surgical trauma. Because of the expansion of auxiliary tools, the incision used in this operation is slightly larger than that in endoscopic surgery, but this does not affect the postoperative management of patients. The patients in this group did not have incision pain and infection after operation, and the average length of hospital stay was similar to that of patients undergoing endoscopic surgery in our hospital. Previous literatures considered that muscle injury was an important factor that affected the healing of surgical incision and incision pain (21), while the channel was established by expanding the tube step by step when CKH-UBO was performed with the aid of channel, which could effectively reduce the injury of neck muscle. The average intraoperative bleeding volume of this group was 32.7 ± 4.1 ml, which was lower than that of the traditional open posterior cervical laminectomy (22), and similar to the results reported in the literature of key hole under some endoscopes (23, 24). Intraoperatively, we found that the main source of bleeding was the intraspinal hemorrhage after the incision of the ligamentum flavum. Under the channel, we can use gelatin sponge and brain cotton for rapid compression hemostasis. In addition, when the UBO is used to remove the bone structure, it has a cavitation effect, which can coagulate and denature the hemoglobin within the cutting range and also play a certain hemostatic role (25, 26). No drainage room was placed in this group of patients after surgery. Lower bleeding volume can not only improve the safety of surgery, but also reduce the difficulty of postoperative patient management. The protection of the local stability of the cervical spine is very important in the key hole technology. If too much damage is done to the facet joint, it may lead to instability of the cervical spine, and then cervical vertigo, neck and shoulder pain and other complications (27, 28). The channel assisted CKH-UBO is used for nerve decompression under direct vision. Sufficient surgical field can facilitate the operator to accurately judge the anatomical position and prevent the decompression range from being too large and affecting the stability. In addition, the precise bone cutting characteristics of the UBO can avoid unnecessary bone structure damage caused by improper operation. In this group of trials, all patients did not have instability of the surgical segment after operation. This result shows that this surgical method can effectively protect the local stability of the cervical spine, and there is no significant difference in ROM of the cervical spine before and after operation, which also reflects the advantage of key hole technology in fully protecting the mobility of the cervical spine compared with anterior cervical spine fixation and fusion surgery.

In terms of nerve damage. When the key hole technology under endoscope uses a grinding drill to remove the bone structure, the grinding head is likely to leak and slip, thus affecting the surrounding nerve tissue, and in serious cases, it will lead to disastrous consequences (29). This is mainly due to the poor stability of the grinding drill when the force direction of the grinding drill is toward or parallel to the spinal cord. Therefore, even if the operator has high proficiency in grinding and drilling, the risk can not be completely avoided. When the UBO is used under the channel, the direction of its force is away from the dural sac. It is carried out in a way similar to the electric curette. It has strong controllability and can completely avoid the physical damage of nerve and blood vessels. In addition, in order to avoid burning of peripheral nerve and blood vessels due to high temperature caused by friction, the traditional grinding drill needs to continuously inject physiological saline locally during the removal of bone structures. However, the UBO generates less heat and has a water spray function, which can effectively reduce the local temperature. These characteristics can effectively reduce the risk of nerve and blood vessel burns (30, 31). However, it should be noted that the local temperature can also gradually increase when the UBO is used for decompression for a long time. Therefore, when the UBO is used for a long time in complex cases, physiological saline should be used intermittently for cooling. The operator should also pay attention to the local temperature changes at all times to avoid skin burns. In this experiment, one patient felt numbness of the affected side of the upper limb after operation and completely relieved 3 days after operation. We considered that the nerve root was stimulated when the nucleus pulposus tissue was removed due to severe nerve compression in this patient. The other enrolled patients had no neurospinal cord injury, indicating that it is very reliable in neuroprotection.

## Conclusion

We believe that the channel assisted CKH-UBO treatment for single segment CSR has short operation time, reliable clinical efficacy, high safety and low complication rate, and is worthy of clinical promotion. However, the number of cases enrolled in this study is small, and there is a lack of long-term follow-up results. It is hoped to be supplemented in future research.

## Data Availability

The raw data supporting the conclusions of this article will be made available by the authors, without undue reservation.
