# Supplementary material for: Clinical effect of channel assisted cervical key hole technology combined with ultrasonic bone osteotome in the treatment of single segment cervical spondylotic radiculopathy
Source: Front Surg. 2022 Oct 17;9:1029028. doi: 10.3389/fsurg.2022.1029028 (PMC9618798; doi:10.3389/fsurg.2022.1029028)
Supplement: Supplementary file 1 [file Table1.docx]

**Table 1 Summary of the baseline data**

| **Characteristics** | **CKH-UBO(n=14)** |
| --- | --- |
| **Age (years)** | 51.1±8.4 |
| **Sex M/F** | 11/3 |
| **Duration of symptoms（months）** | 16.9±7.5 |
| **Surgical location**  C4/5  C5/6  C6/7  **Operating time(min)**  **Blood loss(ml)**  **Incision length(cm)**  **Hospital stay(d)** | 3  7  4  42.2±5.7  32.7±4.1  2.0±0.1  5.6±1.2 |

**CKH-UBO indicates Cervical Key Hole-Ultrasonic Bone Osteotome; n indicates the total number of patients.**

**Table 2 Comparation of the ROM between pre and postoperative**

| **Characteristics** | **Pre-op** | **Post 3m-op** | **Post 1y-op** |
| --- | --- | --- | --- |
| **ROM** | 51.3±3.1^*^ | 50.1±2.6^&^ | 51.2±3.5^#^ |

**ROM indicates Range of motion.**

**The scoring system is used to assess lumbar spine stability. P=0.387 if & is compared with *, P=0.833 if # is compared with *.**

**Table 3 Comparation of the Functional score between pre and postoperative**

| **Characteristics** | **Pre-op** | **Post 3d-op** | **Post 3m-op** | **Post 1y-op** |
| --- | --- | --- | --- | --- |
| **VAS neck**  **VAS upper limb**  **NDI**  **Macnab**  **Excellent**  **Good**  **Fair**  **Poor**  **E and G Rate** | 5.6±1.2^*^  6.2±1.2^#^  36.7±3.5^$^ | 1.6±0.6^&^  1.7±0.7^%^  9.8±2.4^^^ | 1.1±0.7^&^  1.1±0.6^%^  3.9±1.5^^^ | 0.6±0.5^&^  0.6±0.5^%^  1.8±1.0^^^  9  5  0  0  100% |

**VAS indicates Visual Analogue Scale; NDI indicates Neck Disabilitv Index; E indicates Excellent；G indicates Good；pre-op indicates preoperative; post-op indicates postoperative.**

**P<0.001 if & is compared with *, P<0.001 if % is compared with #, P<0.001 if** ^ **is compared with** $
